# Abstract of Discussion

**Published:** 1916-10

**Authors:** 


### ABSTRACT OF DISCUSSION

### ON PAPERS OF DRS. MOOREHEAD, BILLINGS AND IRONS.

Dr. William C. Fisher, New York: There seems to be an idea among the medical and dental professions, and it is developing among the laity, that in order for a dentist to treat roots properly he must have the M.D. degree. No one wants the higher education of our dentist extended more than I do, but there are thousands of dentists in the United States who have only a dental college education, and have been properly trained in dental surgery. There is no excuse for any dentist not doing good root canal work, even if he has only the education which he received from a dental college. While a medical education is of great aid to the dentist in his diagnostic work and in his collaboration with the medical man and in research work, yet I do not wish to see the responsibility lifted from the general dental practitioner who has only a dental degree, and I do not want the public and the medical profession to think that the something that was not given him in the dental college is the reason why we have this poor root work. They have sufficient technical education to do good root work, and if they do not do it, the fault is with the individual man. If a tooth is devitalized and the roots have never been filled, and still the Roentgen ray does not reveal any shaded area or subnormal area beneath the root, that is not an assurance that there is no infection there. If the Roentgen ray shows a devitalized tooth that has not had proper root treatment, then that tooth should be opened and an attempt made to treat it. If the dentist can not go through to the apical foramen of each and every root of that tooth, then that tooth should be extracted. My reason for that is the fact that in a case of arthritis a few weeks ago there were two such teeth, lower molars, on both sides of the jaw, that it had been impossible for myself and one other dentist to get through. I insisted on extraction, and my bacteriologic confrere reported in a few days that he had *streptococcus viridans* from the apexes of both molars. The roentgenograms in this case were read by our most expert men, and they could not find any shading at the ends. The general practitioner was looking for a focus of infection, and we found two areas in those root sockets.

Dr. John E. Nyman, Chicago: Local infections of the mouth which cause systemic derangements may be divided into two classes. First, the open discharging variety, those that produce systemic derangement by reason of their influence on and through the digestive tract, such as discharging pockets of pyorrhea unaccompanied by pain or soreness about the teeth, and resulting in a slow systemic toxemia. Second, the enclosed form of focal infection, producing systemic derangement directly through lymph and blood vessels. The first form of infection may give rise to digestive derangement; the frequent hyperchlorhydrias without definite cause, resulting later, perhaps, in gastric ulcer; or in spastic constriction in the colon, predisposing to acute or chronic subacute attacks of appendicitis. These toxemias are sometimes related to mental derangements. I think Dr. Craig of New York reported some twenty cases of melancholia which showed no improvement until pyorrheal conditions had been eradicated. The other form, that of metastatic infection through the lymph and blood channels, may result in such conditions as chronic arthritis, single or multiple, also in cardiac derangements with an intermitten angina, or cardiac insufficiency. Through my association with men who are in the practice of medicine I can relate a list of diseases which were really definitely relieved, even cured, by correcting the abnormal conditions in the mouth. This list comprises arthritis, cardiac derangement, angina, hyperchlorhydria, spastic constriction of the colon, reflex neuroses, insomnia, and all forms of phobias. My experience has been that many phobias and insomnias have been due to mechanical disturbance by impacted unemptied teeth. Recently I had three cases which have been cleared up by removal of badly impacted teeth. Even if no definite systemic disturbance of any kind is reported coincident with pathologic conditions in the mouth, the continuance of these conditions in due course of time has its effect on the tissues of the body, resulting in a form of cell depreciation, so that the individual is more susceptible to an acute invasion. I am glad Dr. Irons spoke as he did concerning vaccines. There is always danger in the use of vaccines of developing a state of "sensitization" or a form of anaphylaxis. In a subacute chronic arthritis we have not a disease to deal with which is ordinarily dangerous or acutely distressing to the individual, and we may, in administering a vaccine for the purpose of quickly eradicating that condition, "sensitize" the patient so that if some dangerous condition develops through meningitis, diphtheria or tetanus which requires the administration of a specific serum the injection may result in a fatality.

Dr. W. A. Price, Cleveland: I recently asked the pathologist in a hospital what proportion of the teeth he saw extracted were related, directly or indirectly, with the lesion for which they were extracted, and he said, "Not twenty-five per cent." Again, one of our leading medical pathologists stated that every tooth with a devitalized pulp was a sequestrum. Therefore, devitalized teeth must be extracted. In the researches we have been making in the Research Institute of the National Dental Association we have not been able to sterilize one single canal, so that where there was not a fistula we could not aspirate a culture from beyond that apex after the treatment. More than that, we have never been able to produce a condition in which we could not get the culture from a canal, without a fistula, where there was an apical infection, within six days. This was the case, no matter what medicine was used. We tried everything from sulphuric acid to formalin, and various medicines which we knew should not have been used in ordinary conditions, but which we used as part of the experiment. So far as I am concerned, I can not sterilize the area beyond the tooth apex by any means, but I do believe we dentists can so change the balance of safety for that patient that we will reduce the infection and give nature a chance to destroy that infection.

Dr. Frank Billings, Chicago: I thought I stated distinctly that the reaction of pathogenic organisms invading the tissues anywhere would produce a serous, a fibrinous or a purulent exudate, and I named some other conditions. Apparently the difference in the reaction of the tissues depends on the virulence of the specific infectious agent. In the one event it is virulent enough to cause a positive chemotaxis, and leukocytes wander through the local tissues and if in sufficient numbers, we call it suppuration. If less virulent, it allows less reaction, fibrinous in character, and walls off the micro-organism. In a less virulent type and on the surface of the serous and mucous membranes, a serous exudate is caused, which may wash away the infectious agents. Therefore, a degree of pathogenesis, as we understand it, is the thing which characterizes the reaction which takes place in these tissues. Then, when we go further, and with the members of the streptococcus group attempt to say that this one does this and that one does the other thing, because it produces certain characteristic conditions on a culture medium, we are going far astray. Schottmueller classified the streptococci which are pathogenic for man. He found that one type destroyed the blood corpuscles of the medium; therefore, was hemolytic; another one produces mucous conditions, and he called it the *streptococcus mucosus;* another one produces green halos about the colonies, with some destruction of the red cells, and he called it *streptococcus viridans.* The theory that the *streptococcus viridans* is the least virulent of all streptococci can not hold, because the different strains which have the power to produce green in the culture medium have not the same virulence or pathogenicity. Nor can we say that the one that destroys blood is always a pusproducing one, because the *streptococcus erysipelatus* does not usually produce suppuration. It has an affinity for the subcutaneous tissues and does not usually produce pus. *Streptococcus viridans,* having its source in some focal infection, may have that tropism which I mentioned, which makes it pathognic for the endocardium. If the endocardium is scarred by an old disease, it may therefore make a better soil, and enormous vegetations are produced on the valves. There will be a constant bacteremia. It will immuinize itself against the host. Another type of *streptococcus viridans* may produce changes in joints and muscles and have no affinity whatever for the endocardium. All these organisms are the so-called endotoxic bacteria, and we are in doubt as to whether the toxin is the specific agent that produces the tissue reactions, whether it is the protein combined with the toxin that produces the reaction, or whether it is some split product of the protein, as Vaughan believes. There is something specific about it, otherwise we would not have the one producing pus, the other an erysipelas, the other infection of the heart, another acute rheumatism, another chronic affections of the muscles and joints. Therefore, when you ask about vaccines you must first of all make the bacteriologic diagnosis, to know that you are using the specific agent that causes the disease. We have attempted to study the possible value of so-called autogenous vaccines. In over five hundred cases of chronic arthritis we have compared the results of the vaccinated patients with those not vaccinated but receiving the same hygienic treatment, and the result was apparently just as good without as with the vaccines. Furthermore, where one would sometimes note a local reaction from a vaccine, the same reaction would also result from a non-specific protein.

Swallowed bacteria meet with resistance of the intact mucous membrane throughout the intestinal canal. They meet with destructive influences of the gastric juice, and it is a question always as to how many pass on into the intestine. To say that they are destroyed there and the toxins absorbed to produce general disease is going away back to our old ideas of autoinfection or autointoxication. If intestinal infection does occur, it must mean that the organisms are viable enough, resistant enough, to pass the barrier of the stomach, reach the intestine and pass into the mucosa, and from that into the lymphoid structures of the mesentery, and there form a new focus. Practically, general infection occurs hematogenously or lymphogenously. Even if the lymphogenous infection occurs first, the infectious agents pass from the lymph nodes into the blood stream, and finally, therefore, the route of infection is hematogenous. We must get away from our old ideas of toxin absorption from a focal infection or from the gastro-intestinal tract as causing general disease.

Finally, when we come to therapy dentists need not feel at all mortified about what they do. It is not a question always, I think, of technic in dental practice. It is a question as to how you are going to get rid of existing conditions. The condition of the host is important, not only in primary infection, but in the continued infections as well. If the dentist does not recognize that the condition of that patient whose mouth he is treating requires that he should go to some doctor who is wise enough to build up his natural defenses by hygienic measures, then the dentist is not doing his part for the patient. If the doctor to whom the patient is sent by the dentist, because he finds focal infection about the jaws, does not recognize that focal infection is more dangerous in individuals who are debilitated and does not do his part, he should not blame the dentist because he does not eradicate the focus.

Dr. F. B. Moorehead, Chicago: The one vital thing that the dentist must face in this matter of chronic infections is this: He alone is responsible for the removal of infection from the mouth. It is a demonstrable fact that certain well defined infections may result from foci about the teeth. Whether at the time the patient may be sick or well there is always a margin of doubt, a margin of contingent disability that the patient must be guarded against. The dentist, therefore, must make certain that the tissues have been placed in a state of health before the patient leaves his hands. In the hands of highly trained men certain conservative measures may be undertaken, but the general practitioner, the average man, may not assume the role of a specialist. The tissues of the mouth should not be looked on with an unwarranted suspicion. Infections about the teeth should be regarded in the same light as infections involving the appendix, gallbladder or the tonsil. It would seem to be a matter of plain, ordinary common sense to remove infection wherever it may be found.—*Journal A. M. A.*, September, 1916.

